# SmokefreeTXT for Homeless Smokers: Pilot Randomized Controlled Trial

**DOI:** 10.2196/13162

**Published:** 2019-06-04

**Authors:** Travis P Baggett, Claire McGlave, Gina R Kruse, Awesta Yaqubi, Yuchiao Chang, Nancy A Rigotti

**Affiliations:** 1 Tobacco Research and Treatment Center Division of General Internal Medicine Massachusetts General Hospital Boston, MA United States; 2 Institute for Research, Quality, and Policy in Homeless Health Care Boston Health Care for the Homeless Program Boston, MA United States; 3 Harvard Medical School Boston, MA United States; 4 Harvard TH Chan School of Public Health Boston, MA United States; 5 Boston University School of Medicine Boston, MA United States

**Keywords:** homeless persons, cigarette smoking, smoking cessation, text messaging

## Abstract

**Background:**

Homeless smokers want to quit smoking but face numerous barriers to doing so, including pervasive smoking among peers and a lack of social support for quitting. An SMS (short message service) text messaging intervention could address these challenges by providing virtual daily support for homeless smokers who are trying to quit but coping with multiple triggers to smoke.

**Objective:**

This study aimed to assess whether a free SMS text messaging program, added to evidence-based pharmacotherapy and counseling, improved smoking abstinence among homeless adult smokers.

**Methods:**

From October 2015 to June 2016, we conducted an 8-week pilot randomized controlled trial (RCT) of nicotine patch therapy and weekly in-person counseling with (n=25) or without (n=25) SmokefreeTXT, a free SMS text messaging service administered by the National Cancer Institute (NCI) at Boston Health Care for the Homeless Program. All participants were provided with a mobile phone and a 2-month prepaid voice and text plan at no cost. SmokefreeTXT enrollees were sent 1 to 5 automated SMS text messages daily for up to 8 weeks and could receive on-demand tips for managing cravings, mood symptoms, and smoking lapses. The primary outcome was smoking abstinence, defined as an exhaled carbon monoxide count of <8 parts per million, assessed 14 times over 8 weeks of follow-up, and analyzed using repeated-measures logistic regression with generalized estimating equations. Other outcomes were use of SmokefreeTXT, assessed by data obtained from NCI; perceptions of SmokefreeTXT, assessed by surveys and qualitative interviews; and mobile phone retention, assessed by self-report.

**Results:**

Of the SmokefreeTXT arm participants (n=25), 88% (22) enrolled in the program, but only 56% (14) had confirmed enrollment for ≥2 weeks. Among 2-week enrollees, the median response rate to interactive messages from SmokefreeTXT was 2.1% (interquartile range 0-10.5%). Across all time points, smoking abstinence did not differ significantly between SmokefreeTXT and control arm participants (odds ratio 0.92, 95% CI 0.30-2.84). Of SmokefreeTXT enrollees who completed exit surveys (n=15), two-thirds were very or extremely satisfied with the program. However, qualitative interviews (n=14) revealed that many participants preferred in-person intervention formats over phone-based, found the SMS text messages impersonal and robotic, and felt that the messages were too frequent and repetitive. Only 40% (10/25) of SmokefreeTXT arm participants retained their study-supplied mobile phone for the 8-week duration of the trial, with phone theft being common. Storing and charging phones were cited as challenges.

**Conclusions:**

SmokefreeTXT, added to nicotine patch therapy and in-person counseling, did not significantly improve smoking abstinence in this 8-week pilot RCT for homeless smokers. SMS text messaging interventions for this population should be better tuned to the unique circumstances of homelessness and coupled with efforts to promote mobile phone retention over time.

**Trial Registration:**

ClinicalTrials.gov NCT02565381; https://clinicaltrials.gov/ct2/show/NCT02565381 (Archived by WebCite at http://www.webcitation.org/78PLpDptZ)

## Introduction

### Background

An estimated 2.3 to 3.5 million people experience homelessness each year in the United States [1], with over 553,000 homeless on any given night [2]. The prevalence of smoking among homeless adults is about 4 times higher than in the general population [3-8], contributing to over 2-fold higher rates of lung cancer [9] and 3- to 5-fold higher rates of tobacco-attributable death [10].

Studies have consistently demonstrated that homeless smokers want to quit smoking [4,11-14], but prior randomized controlled trials (RCTs) have not yet revealed the optimal approach to promoting smoking cessation in this vulnerable population. The largest such study to date examined the effect of motivational interviewing to promote nicotine patch adherence, which was not significantly better than control in producing smoking abstinence at 6 months [15].

The pervasiveness and social acceptability of smoking in the setting of homelessness [14,16] may contribute to low social support for quitting and frequent cues to continue smoking. In a survey of homeless smokers in Dallas, participants reported contact with a mean of 43 other smokers each day [17]. Treatment strategies must contend with this and other prosmoking influences that homeless smokers encounter daily outside the domain of a traditional health care setting.

Smoking cessation interventions delivered via a mobile device might enhance traditional interventions by providing virtual daily support to homeless smokers who are trying to quit but coping with multiple social and environmental triggers to relapse. Mobile phone possession is common among homeless people [18], and mobile technologies have been used to deliver appointment reminders to homeless veterans [19] and to collect ecological momentary assessment data from homeless smokers making a quit attempt [20,21]. Although SMS (short message service) text messaging interventions for smoking cessation have demonstrated efficacy across a range of settings and populations [22-33], no studies to our knowledge have examined SMS text messaging interventions for homeless smokers.

### Objective

To address these gaps in the literature, our objective was to assess whether a free SMS text messaging program, SmokefreeTXT, improved smoking abstinence among homeless adult smokers when added to evidence-based pharmacotherapy and in-person counseling. Developed by the National Cancer Institute (NCI), SmokefreeTXT provides around-the-clock and on-demand support for adults who want to quit smoking [34] through SMS text messaging content that incorporates a variety of behavior change techniques [35]. Although SmokefreeTXT is not targeted specifically to homeless or low-income smokers, its accessibility and potential for immediate dissemination, if effective, made it appealing for testing in a highly impoverished population.

## Methods

### Study Design

We conducted a 3-arm, parallel group, nonblinded, 8-week pilot RCT that tested 2 separate smoking cessation interventions: (1) SMS text messaging delivered via SmokefreeTXT to support smoking abstinence and (2) financial incentives for smoking abstinence against (3) a shared control condition consisting of counseling and nicotine replacement therapy. We originally planned a 2-arm trial examining financial incentives against control treatment. However, additional funding enabled us to add a third study arm examining SMS text messaging relative to the same control condition. We prespecified a plan to analyze the SMS text messaging and financial incentives interventions separately because of the pilot nature of the trial and the differing rationales for each approach. This paper compares the effect of the SMS text messaging intervention with the control condition. The financial incentives findings are published elsewhere, as is a detailed description of the participants and setting, enrollment and randomization procedures, and baseline and follow-up assessments [36]. The following sections summarize those elements while emphasizing the details of the SMS text messaging intervention. The study protocol was approved by the Partners Human Research Committee and registered with ClinicalTrials.gov (NCT02565381) before commencement. All study procedures occurred from October 2015 to June 2016 at Boston Health Care for the Homeless Program (BHCHP) headquarters.

### Enrollment and Randomization

Participants were recruited through in-person advertisement in the BHCHP lobby, flyers posted in BHCHP clinics, and referrals from BHCHP clinicians. Eligibility criteria were aged 18 years or older, lifetime smoking of 100 or more cigarettes [37] with current smoking of 5 or more cigarettes/day, verified by an exhaled carbon monoxide (CO) level of ≥8 parts per million (ppm) [38], readiness to quit smoking within the next month, current homelessness, and self-reported English proficiency. We defined current homelessness as usually staying in an emergency shelter, transitional shelter, abandoned building, place of business, car or other vehicle, church or mission, hotel or motel, or anywhere outside during the past 7 days, or if currently in a residential treatment program, in the 7 days before program entry. In addition, individuals were considered currently homeless if they usually stayed in somebody else’s place in the past 7 days because of not having their own place to stay. This definition is generally concordant with the US federal definition of homelessness [39] and identical to the definition that we and others have used in prior studies [40-44].

Exclusion criteria were current pregnancy, past-month use of any smoking cessation medication, prior serious adverse reaction to the nicotine patch, myocardial infarction or undiagnosed chest pain in the past 2 weeks, and inability to read a sentence written at a Flesch-Kincaid 4th-grade level. Participants were not excluded because of active substance use or mental illness.

We used a multistep enrollment process described elsewhere [36] to ensure that participants sufficiently understood the study and were committed to participating. All participants provided written informed consent to participate. Enrolled participants were randomized 1:1:1 to the SmokefreeTXT arm, financial incentives arm, or control arm. The allocation sequence was computer-generated in random permuted blocks and concealed from study staff.

### Baseline Measures

Participants completed a baseline assessment of self-reported sociodemographic, health, and smoking characteristics at the time of enrollment. Sociodemographic measures included age, sex, and race and ethnicity. We asked participants to rate their general health status. We used the Addiction Severity Index-5th edition [45], which has been validated in homeless populations [46-48], to assess past-month alcohol use, drug use, and psychiatric symptom severity, with a problem in each of these domains based on score cut-offs described elsewhere [49]. Smoking characteristics included current cigarette dependence (assessed with the Fagerstrom test of nicotine dependence, FTND; range 0-10) [50], confidence to quit and perceived importance of quitting (each assessed using 10-point scales), and previous smoking cessation attempts.

### Mobile Device

Participants in all study arms received a mobile phone with a QWERTY keyboard and a 2.4-inch display (AT&T ZTE Z432; retail US $29.99 each). The phones were activated by study staff at the time of randomization and loaded with a prepaid 2-month voice and text plan. We provided the same mobile device and plan to all participants in all arms, regardless of whether they already had a cell phone of their own, to deliver brief reminder phone calls and/or SMS text messages about upcoming study appointments, ensure uninterrupted mobile phone service for everyone, and standardize the end-user interface. Mobile devices and plans were provided at no cost to participants. Participants were informed that they would be given only 1 device to last the duration of the study. To incentivize phone retention, participants were allowed to keep their study phones if they still had them at the end of the trial but were told that they would need to cover the cost of any additional usage beyond the 2-month study period.

### Control Arm

Participants assigned to the control arm were offered 8 weeks of nicotine patch therapy and weekly in-person counseling.

#### Nicotine Patch Therapy

Nicotine patches were distributed in 1-week allotments at no cost to participants. Participants who smoked 10 or more cigarettes per day were started on 21 mg/day patches and tapered to 14 mg/day at week 6. Participants who smoked less than 10 cigarettes per day were given 14 mg/day patches throughout the 8-week study.

#### In-Person Counseling

Participants were offered 8 weekly in-person counseling sessions lasting up to 15 min each. The counseling curriculum was developed by our study team in collaboration with a certified Master Tobacco Treatment Specialist (TTS) and was structured around the American Lung Association “Freedom from Smoking” program theme of addressing the physical, mental, and social aspects of tobacco addiction [51]. Counseling sessions incorporated elements of motivational interviewing and cognitive behavioral therapy and were tailored to the unique needs and circumstances of people experiencing homelessness. Before the trial, the study counselor completed a 9-module Web-based training course on basic skills for working with smokers, 6 hours of case-based didactics conducted by a certified Master TTS, 6 hours of observing a certified TTS provide counseling to smokers, and 3 hours of observing a clinician interact with homeless patients.

### SmokefreeTXT Arm

Participants assigned to the SmokefreeTXT arm received nicotine patch therapy and in-person counseling in a fashion identical to the control arm. In addition, participants in this arm were offered assistance with enrolling in SmokefreeTXT. At enrollment, SmokefreeTXT prompted participants to set a quit date within the next 2 weeks, although the date could be adjusted later if desired. The program then sent 1 to 5 automated SMS text messages daily that provided encouragement, advice, and tips for quitting smoking. Message frequency and content varied according to where participants were in relation to their specified quit date as well as whether and how long they abstained from smoking following their quit date. SmokefreeTXT message content was updated periodically by NCI and was not tailored to homeless or low-income individuals. A behavior change technique analysis of the SmokefreeTXT library conducted contemporaneously with our study found that 14 of 16 behavioral technique groups outlined in the Behaviour Change Technique Taxonomy version 1 were present in SmokefreeTXT, with the most prevalent being feedback and monitoring, natural consequences, social support, and shaping knowledge [35]. Most SmokefreeTXT messages were unidirectional, but beginning on the quit date, a subset (approximately 23%) of messages were interactive in nature and solicited brief participant responses (eg, “Feelings can be a smoking trigger. If you feel cranky or grouchy, it is only temporary, so stay strong. How is your mood? Reply: GOOD, OK, or BAD”). In addition, participants could spontaneously text keywords (“crave,” “mood,” or “slip”) at any time to signal the need for automated supportive messaging around the management of cravings to smoke, distressing mood symptoms, or lapses in smoking abstinence. Participants could unsubscribe from SmokefreeTXT at any time. If participants lost their study-issued mobile phone but wished to continue using SmokefreeTXT and had another mobile device of their own, we offered to assist them in re-enrolling in SmokefreeTXT under their other phone number.

### Assessment Procedure

Following randomization, all participants were asked to make 14 assessment visits over 8 weeks: thrice weekly during weeks 1 and 2, twice weekly during weeks 3 and 4, and once weekly during weeks 5 and 8. At each visit, study staff measured participants’ exhaled CO levels using a Micro+ Smokerlyzer CO monitor (Bedfont Scientific Ltd; Maidstone, Kent, United Kingdom). The intensive nature of the follow-up assessment scheme ensured equal attention to all study arms, as the financial incentives intervention required frequent abstinence monitoring for maximum effect [52]. Control and SmokefreeTXT arm participants received US $10 payments onto study-supplied debit cards for each assessment visit they attended, regardless of whether they were abstinent. Participants in both arms received brief SMS text message reminders from study staff about their assessment visit appointments, and they also received public transportation tickets to facilitate attendance.

### Outcomes

#### Smoking Outcomes

The prespecified primary study outcome was a repeated measure of brief smoking abstinence, defined as an exhaled CO <8 ppm [38] and assessed 14 times over 8 weeks. We used exhaled CO rather than nicotine metabolites to define smoking abstinence because the latter can be affected by nicotine replacement therapy [38], which was provided to all participants. We did not incorporate self-report into the primary outcome definition because the financial incentives arm provided abstinence-contingent financial rewards that created the potential for differential misreporting of smoking status. In a sensitivity analysis, we combined exhaled CO data with self-reported past-week smoking behavior to create 2 alternative abstinence outcomes, each assessed 8 times over 8 weeks: (1) Past 7-day cigarette abstinence: CO <8 ppm and last smoked all or part of a cigarette ≥7 days ago and (2) Past 7-day puff abstinence: CO <8 ppm and last smoked even a puff of a cigarette ≥7 days ago. Other smoking-related outcomes included past-month 24-hour quit attempts, assessed at 4 and 8 weeks.

#### Attendance

We assessed the number of study visits and the number of in-person counseling sessions attended by participants in each study arm.

#### SmokefreeTXT Engagement and Perceptions

We used a mixed methods approach to assess SmokefreeTXT arm participants’ engagement with and perceptions of the texting program.

First, we obtained data from NCI to confirm whether participants enrolled in SmokefreeTXT, whether and when they unenrolled from the program, and whether they responded to interactive SMS text messages. Data on participants’ spontaneous use of SmokefreeTXT keywords (“crave,” “mood,” or “slip”) were not available.

Second, SmokefreeTXT arm participants who attended the final study visit were asked to complete a survey containing Likert-type items assessing their satisfaction with the SMS text messaging program, the perceived importance of the program in helping them to quit, the applicability of the messages to their everyday lives, and the likelihood that they would recommend the program to others.

Finally, 14 weeks after the study opened for enrollment, we added an in-person qualitative exit interview to assist in contextualizing and explaining the RCT results. Participants who had already completed the study were retroactively contacted about participating in the interview, whereas the remainder were prospectively offered the opportunity to complete the exit interview on or shortly after their final study visit. Study staff followed a semistructured interview guide that prompted all participants to reflect on their experience with each of the intervention components as well as with the mechanics of the study itself to inform future work.

SmokefreeTXT arm participants were additionally asked open-ended questions about whether SmokefreeTXT was useful in helping them to quit or cut back smoking, what they liked most about the SMS text messages they received, the frequency of the messages, and what they would change about the program. Participants received US $10 for completing the exit interview.

#### Mobile Phone Retention and Perceptions

The potential benefits of an SMS text messaging program can be realized only if participants retain a mobile device capable of receiving SMS text messages for the intended duration of the program and feel comfortable using that device to achieve health-related goals. To assess mobile phone retention, we asked participants at each assessment visit whether they still had their study-issued mobile phone. At the final study visit, we asked participants to rate their level of satisfaction with using a mobile phone to participate in a research study. In addition, the qualitative exit interviews described above asked participants about their perceptions of the features and benefits of their study-issued mobile phone, particularly in relation to their own phone if they had one, to better understand end-user mobile device preferences.

### Analysis

#### Smoking Outcomes

We used repeated-measures logistic regression with generalized estimating equations (GEEs) to estimate the overall effect of SmokefreeTXT on the primary outcome of brief smoking abstinence (exhaled CO <8 ppm) across 14 measurements and on alternative definitions of smoking abstinence that combined exhaled CO data with self-reported smoking behavior (past 7-day cigarette and puff abstinence) across 8 weekly measurements. Our main analysis (1) included only treatment effect in the GEE model, (2) assumed that those with missing abstinence data at any given time point were nonabstinent, and (3) was based on the intention to treat principle. In sensitivity analyses, we examined the impact of (1) multivariable adjustment for age, sex, race, nicotine dependence score, and baseline alcohol use, drug use, and psychiatric symptom severity scores; (2) multiple imputation of missing abstinence outcomes [53,54] based on nonmissing abstinence values in addition to age, sex, race/ethnicity, nicotine dependence score, and baseline alcohol use, drug use, and psychiatric symptom severity scores; and (3) reassessing the effect of SmokefreeTXT among those who enrolled in the program for at least 2 consecutive weeks (the point at which a quit date had to be set and interactive messaging began) to estimate the effect of treatment on the treated. We used repeated-measures linear regression with GEE to estimate the effect of SmokefreeTXT on past-month 24-hour quit attempts across 2 measurements (4 and 8 weeks).

#### Power Analysis

Before the start of the study, we used simulated data to estimate statistical power based on assumptions derived from the largest smoking cessation RCT for homeless individuals [15] and the most recent Cochrane systematic review of mobile phone–based interventions for smoking cessation [55]. The power analysis was based on the primary outcome of brief smoking abstinence measured 14 times over 8 weeks. The sample size (N=25 per arm) was dictated by the financial resources available for this pilot study. We assumed a similar pattern of missing data across both study arms, with 90% attendance at the first assessment, decreasing to 50% at the study midpoint, and staying flat thereafter until increasing to 80% at the last assessment. For the control arm, we assumed that CO-defined abstinence would be 10% at the first assessment and 8% thereafter. For the SmokefreeTXT arm, we assumed 25% abstinence at the first assessment, decreasing to 15 to 16% abstinence over subsequent visits. On the basis of these assumptions, we had 82% power to detect the specified differences in brief smoking abstinence.

#### Other Quantitative Analyses

We compared study visit attendance and counseling session attendance between the 2 arms using Wilcoxon tests.

We combined enrollment and unenrollment data from SmokefreeTXT with participant-confirmed mobile phone possession dates to estimate participants’ actual exposure time to the program (ie, dates of SmokefreeTXT exposure during which cell phone possession could be confirmed). Among those with confirmed enrollment in SmokefreeTXT for at least 2 consecutive weeks, we calculated individual-level response rates to interactive SMS text messages by dividing the number of times a participant replied to an interactive message by the total number of interactive messages sent by the program during time periods of confirmed phone possession.

We used descriptive statistics to present the percentages of SmokefreeTXT arm participants who were “very” or “extremely” satisfied with the program, who rated the program as “very” or “extremely” important for quitting, who were “very” or “extremely” likely to recommend the program to others, and who “agreed” or “strongly agreed” that the messages were applicable to their lives. We also descriptively examined mobile phone retention duration as well as the proportion of SmokefreeTXT arm participants who were “very” or “extremely” satisfied with using a mobile phone for a research study.

#### Qualitative Analyses

Exit interviews were audio-recorded and transcribed verbatim by a member of the study staff, and the portions related to SmokefreeTXT and mobile phone usage were extracted for content analysis [56]. Using an inductive approach [57], 2 study staff members independently coded the transcripts. Coding was performed at the sentence level, and multiple coding was permitted. During iterative team meetings, major and minor themes were identified, refined, and organized into a hierarchical thematic framework that guided subsequent iterations of coding until all text had been categorized with an overall inter-rater reliability of kappa=.80. The overall kappa was calculated as the average kappa for all themes, with sources and themes weighted equally.

We used SAS 9.4 (SAS Institute) to conduct quantitative analyses and NVivo 10 (QSR International) to conduct qualitative analyses. Inferential analyses of smoking outcomes used a 2-sided significance level of 0.05.

## Results

### Screening, Enrollment, and Randomization

**Figure 1:**
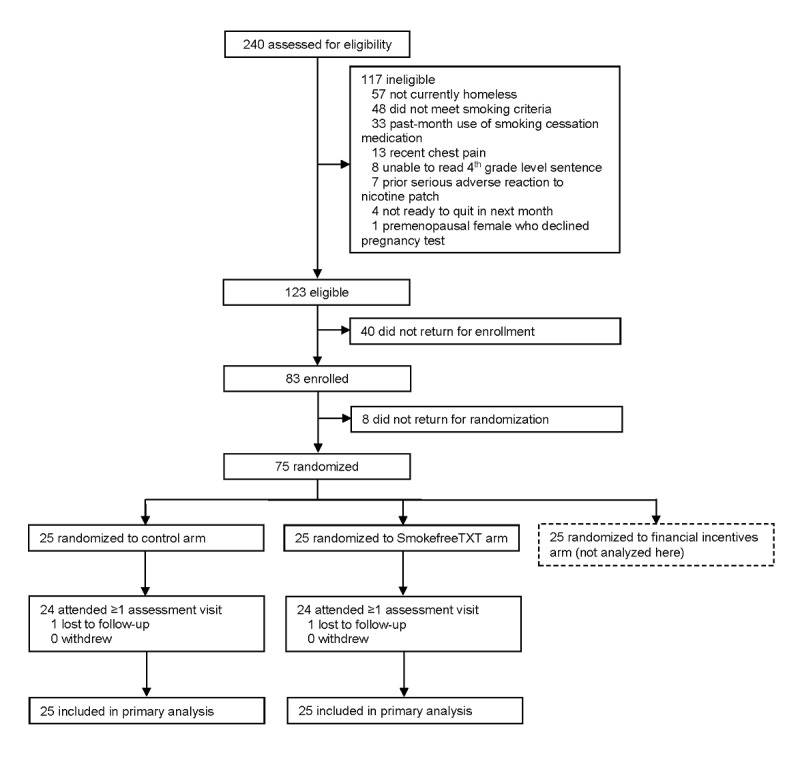
Consolidated Standards of Reporting Trials flow diagram. Sum of exclusion reasons totals greater than 117 because individuals could be ineligible for more than one reason. Smoking inclusion criteria were: (1) lifetime smoking of ≥100 cigarettes, (2) current daily smoking of ≥5 cigarettes per day, and (3) exhaled carbon monoxide level of ≥8 parts per million on 2 separate occasions.

A total of 83 (67%) of 123 eligible individuals enrolled in the study and completed the baseline assessment (Figure 1). A total of 8 enrollees (10%) did not return for randomization; these participants reported higher baseline confidence to quit (*P*=.01) than the 75 participants who returned for randomization, but they did not differ significantly in other ways. The remainder of the results focus on the 50 participants randomized to the control arm (n=25) or SmokefreeTXT arm (n=25).

**Table 1:** Characteristics of study participants (n=50).

Characteristics			All	Control (n=25)	SmokefreeTXT (n=25)
**Sociodemographic**					
	Age, years, mean (SD)		45.6 (9.3)	45.1 (9.6)	46.1 (9.2)
	Female, n (%)		29 (58)	14 (56)	15 (60)
	**Race/ethnicity, n (%)**				
		White, non-Hispanic	20 (40)	10 (40)	10 (40)
		Black, non-Hispanic	19 (38)	9 (36)	10 (40)
		Hispanic	7 (14)	5 (20)	2 (8)
		Other	4 (8)	1 (4)	3 (12)
**Health**					
	Fair or poor health, n (%)		24 (48)	13 (52)	11 (44)
	Alcohol problem, n (%)		10 (20)	3 (12)	7 (28)
	Drug problem, n (%)		29 (59)	14 (58)	15 (60)
	Psychiatric problem, n (%)		30 (60)	15 (60)	15 (60)
**Smoking**					
	Cigarettes per day, mean (SD)		15.8 (6.4)	16.2 (6.3)	15.4 (6.7)
	Nicotine dependence (0-10), mean (SD)		5.0 (1.9)	5.1 (1.8)	4.9 (2.0)
	Past quit attempts, median (IQR^a^)		3 (1-5)	2 (1-4)	3 (2-6)
	Quitting importance (1-10), mean (SD)		8.9 (1.5)	8.8 (1.5)	8.9 (1.6)
	Quitting confidence (1-10), mean (SD)		6.4 (2.3)	6.8 (2.2)	5.9 (2.4)

^a^IQR: interquartile range.

### Baseline Characteristics

The mean age was 45.6 years, 58% of participants (n=29) were female, and 60% (n=30) were non-white (Table 1). Almost half (48%) rated their health as fair or poor. Considerable proportions had a current alcohol use problem (20%), current drug use problem (59%), or current psychiatric problem (60%). Participants smoked an average of 15.8 cigarettes per day and had a mean FTND score of 5.0.

### Attendance

Participants attended a median of 10 (interquartile range, IQR 7-12) of 14 possible assessment visits, with no significant difference between arms (*P*=.72). A total of 96% of participants attended at least one assessment visit, and 76% attended at least half of the assessment visits. Participants attended a median of 1 (IQR 0-3) of 8 possible in-person counseling sessions, with no significant difference between arms (*P*=.77).

### SmokefreeTXT Uptake and Engagement

A total of 22 (88%) of the 25 SmokefreeTXT arm participants enrolled in SmokefreeTXT; the remaining 3 declined to enroll but still participated in the study. Median confirmed exposure to SmokefreeTXT was estimated at 25 days (IQR 5-50 days), with 56% (n=14) being enrolled for at least 2 consecutive weeks. Among those 14 participants, the median response rate to interactive messages from SmokefreeTXT was 2.1% (IQR 0-10.5%).

### Smoking Outcomes

**Figure 2:**
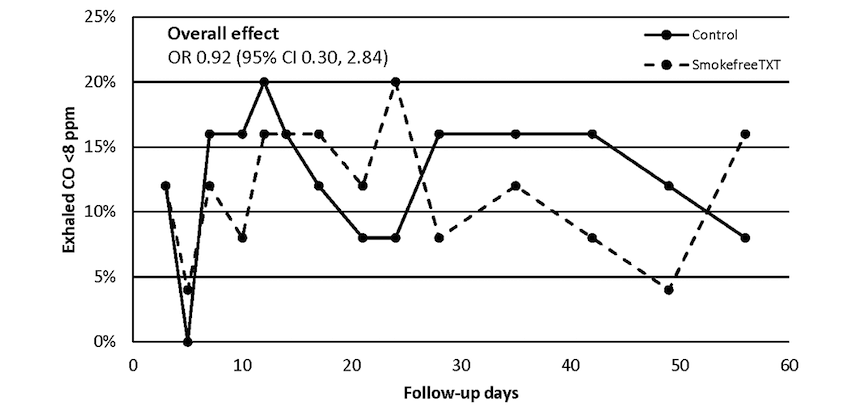
Brief carbon monoxide–defined smoking abstinence by study arm. CO: carbon monoxide; OR: odds ratio; ppm: parts per million.

Across the 14 follow-up visits, brief smoking abstinence (exhaled CO <8 ppm) did not differ significantly between the SmokefreeTXT and control arms (ranges 4-20% vs 0-20%; overall odds ratio [OR] 0.92, 95% CI 0.30-2.84; Figure 2). The treatment effect estimate was not substantively altered by either multivariable adjustment (OR 0.96, 95% CI 0.32-2.95) or multiple imputation of missing smoking status data (OR 1.06, 95% CI 0.72-1.58). Brief smoking abstinence among the 14 participants who were enrolled in SmokefreeTXT for at least 2 consecutive weeks did not differ significantly from control arm abstinence (OR 1.06, 95% CI 0.48-2.37).

When combining exhaled CO data with self-reported past-week smoking behavior over 8 weekly measurements (Table 2), there were no statistically significant differences between the SmokefreeTXT and control arms in either 7-day cigarette abstinence (range 0-12% vs 0-12%; OR 0.65, 95% CI 0.12-3.45) or 7-day puff abstinence (range 0-4% vs 0-4%; OR 2.01, 95% CI 0.19-21.1).

Across 2 monthly measurements, SmokfreeTXT and control arm participants did not differ significantly in past-month 24-hour quit attempts (mean difference –0.06, 95% CI –0.96 to 0.83).

**Table 2:** Seven-day smoking abstinence by study arm. Individuals with missing carbon monoxide or self-reported smoking data are assumed not to meet the criteria for abstinence. Repeated-measures logistic regression with generalized estimating equations demonstrated no statistically significant overall differences between the SmokefreeTXT and control arms for either abstinence measure (see text).

Study week	Exhaled CO^a^ <8 ppm^b^ *and* last cigarette ≥7 days ago		Exhaled CO <8 ppm *and* last puff ≥7 days ago	
	Control, n (%)	SmokefreeTXT, n (%)	Control, n (%)	SmokefreeTXT, n (%)
1	1 (4)	1 (4)	0 (0)	1 (4)
2	3 (12)	2 (8)	1 (4)	0 (0)
3	0 (0)	0 (0)	0 (0)	0 (0)
4	2 (8)	1 (4)	0 (0)	0 (0)
5	2 (8)	3 (12)	0 (0)	1 (4)
6	2 (8)	1 (4)	0 (0)	0 (0)
7	1 (4)	0 (0)	0 (0)	0 (0)
8	1 (4)	0 (0)	0 (0)	0 (0)

^a^CO: carbon monoxide.

^b^ppm: parts per million.

### SmokefreeTXT Perceptions

Of SmokefreeTXT enrollees who attended the final study visit (n=15), 67% were very/extremely satisfied with the program (0% not at all satisfied), 53% rated the program as very/extremely important in helping them to quit (0% not at all important), 93% were very/extremely likely to recommend the program to others (0% not at all likely), and 60% agreed or strongly agreed that the messages were applicable to their lives (0% strongly disagree).

Qualitative interviews (n=14) revealed more nuanced feedback on SmokefreeTXT. Major themes and representative quotes are displayed in Table 3. With respect to the program format, almost all interviewees expressed a preference for in-person rather than phone-based support (“I’d rather come in and talk.”). Indeed, 1 participant cited this as the reason they did not want to sign up for SmokefreeTXT (“Well I just wanted to do it in person instead of on the phone.”). Some felt that the SMS text messages were too impersonal (“Like a robot, everyone gets the same text.”). Views on the interactive aspects were mixed; some admitted to making little use of this feature, whereas others liked it and wanted more. The timing of messages was viewed as important, but many felt that there were too many SMS text messages overall (“They always came poppin’ in all the time.”). With respect to message content, some users found the SMS text messages informative and useful, although many reported that the content was too repetitive (“Different wordings, same thing.”). Occasional interviewees commented on memorable SMS text messages containing recommendations that were at odds with their living circumstances (“There’s one that said ‘Take a bath’...and I’m like ‘Really?... This is a homeless study folks.’”). Participants generally found the message tone to be encouraging, although 1 participant described having a strongly negative reaction to some messages (“I got mad at them texts.”).

**Table 3:** Qualitative findings related to SmokefreeTXT.

Thematic domains		Findings and sample quotes
**Program format**		
	Comparison with in-person format	Universal preference for in-person over phone-based counseling: “I’d rather come in and talk. … I like to do things in person.” “I’d rather just be, up in person. …So that way you can see my expressions, x, y, z.” “I’d love it in person so we can see eye to eye.”
	Automation	Format felt robotic and impersonal to some: “They’re kind of, like, impersonal, you know? Like a robot, everyone gets the same text.”
	Interactiveness	Some made little use of available interactive features: “Um, I never, I don’t think I’ve ever texted them back.” “I responded a couple of times. A lot of times I started to um, kind of honestly disregard it.”
		Others liked interactive features but wanted more: “The interactive stuff is good. …If they had more of that I think people would get involved more.”
	Frequency of messages	Many felt there were too many: “…they always came poppin’ in all the time. Like ‘ring ring ring.’” “No more than once a day. That’s just crazy. What you got to talk about?”
		Some found the frequency appropriate or wanted more: “I think I needed more.”
	Timing of messages	Viewed as important; desire for customizing timing: “Earlier in the day, morning, I would change them to the morning instead of evening/afternoon.”
**Program content**		
	Usefulness for quitting	Many found them useful: “They kept me going… when I was getting ready to smoke it ‘binged’ then and saying, ‘don’t pick up’ and I would put the cigarette down.”
		Some found them unhelpful: “It was kind of like ‘ok, thank you.’ I’m moving on with my day.” “I mean I read them, but they didn’t really change my… they didn’t really change anything for me.”
	Informativeness	Some learned new information: “I loved it… lot of information of things that I wasn’t aware of, related to smoking.”
		Others reported the information or tips were not new: “Some of them were stuff I already knew.” “It’s like, some of them, I already do that.”
	Applicability	Generally deemed applicable, but non-applicable texts were memorable: “Um, do some laundry type of questions... I was like ‘yeah ok.’” “Like there’s one that said ‘take a bath’ … And I’m like ‘Really? This is a homeless study folks.’”
	Repetitiveness	Many found content repetitive: “A few of them started getting repetitive. I’d think ‘oh dammit I saw that one two days ago.’” “Majority of what they said – different wordings, same thing.”
	Tone	Generally found messages encouraging: “I like the message that they’re trying to send and trying to convey.”
		One found texts critical at times: “I got mad at them texts. …I feel like they were being hard on me in some of the texts. …It was – this one sticks with me – ‘nobody told you it was going to be easy.’ …Sticks with me.”

Of note, SmokefreeTXT arm participants who completed exit interviews (n=14) were less nicotine dependent at baseline (mean FTND score 4.2 vs 5.8; *P*=.04), had better study attendance (median visits 11.5 vs 9.0; *P*=.04), and were more likely to attain the primary outcome of brief CO-defined smoking abstinence (OR 4.41, 95% CI 1.20-16.2) than SmokefreeTXT arm participants who did not complete exit interviews (n=11).

### Mobile Phone Retention and Perceptions

Over three-quarters (77%) of SmokefreeTXT arm participants who attended the final study visit reported being very/extremely satisfied with using a mobile phone to participate in a study. However, median study phone retention was 41 days (IQR 12-57), and only 40% of SmokefreeTXT arm participants retained their phone for the entire 8-week study. Theft was the most commonly reported reason for phone loss. This was reiterated in qualitative interviews, where participants also cited logistical challenges with charging and storing their phones (“Honest to goodness that phone was mostly in my bag...in that bag in my locker at the shelter.”). A total of 76% of SmokefreeTXT arm participants already had another mobile phone before the study, and some of these individuals reported that they preferred their own phone to the study phone (“Most of the time I use my regular cell phone...It’s just what I use and check more often.”). Others preferred the study phone, especially if they did not have their own phone or if their own phone had limited features. Keyboard layout and screen size were cited as important factors in phone usability (“Maybe bigger for the punches...‘cause sometimes when you press one button it presses another one.”).

## Discussion

### Principal Findings and Implications

This is one of very few controlled studies to assess the effect of SmokefreeTXT on smoking outcomes, the first to assess biochemically verified smoking abstinence in SmokefreeTXT users, and the first study of an SMS text messaging intervention for homeless smokers. We found that enrollment in SmokefreeTXT was high, but sustained use of the program was modest, interaction with the program was minimal, and perceptions of the program were mixed. When added to nicotine replacement therapy and in-person counseling, SmokefreeTXT had no significant effect on any measure of smoking abstinence or self-reported quit attempts.

Our findings add to the mixed results seen in prior trials conducted in health care settings of SMS text messaging interventions for smoking cessation [32,58-61]. Our results also extend those of prior observational studies of SmokefreeTXT [62,63] and a related NCI SMS text messaging program designed specifically for veterans [64] demonstrating the challenges of sustaining end-user engagement in this program. End of treatment CO-defined brief smoking abstinence rates in our study were comparable with self-reported abstinence rates in these large-scale, noncontrolled, observational studies [62-64] but were notably lower than self-reported abstinence rates in an emergency department-based pilot study of a multicomponent smoking cessation program that included SmokefreeTXT [65].

Although quantitative scales suggested that the majority of participants were satisfied with SmokefreeTXT, qualitative exit interviews revealed more nuanced perceptions and provided insights about potential reasons for the absence of a treatment effect. Most participants expressed a strong preference for in-person treatment modalities and did not care for the automated and impersonal aspects of an SMS text messaging program, underscoring the importance of personal relationships when working with a highly marginalized population. Although most participants considered the messages to be a good source of support for quitting, several found them to be too frequent and too repetitive. In addition, selected messages struck some participants as being memorably incompatible with their daily lives. As SmokefreeTXT arm participants who completed qualitative interviews were more likely to achieve brief smoking abstinence than those who were not interviewed, these mixed views may represent a best-case scenario of end-user experience with the program. Altogether, these findings suggest that SMS text messaging interventions for homeless individuals might be more successful if the delivery format were more customizable and the content better targeted to the unique circumstances of homelessness. Such changes might enhance SMS text messaging program engagement, which appears to be an important determinant of treatment response in other settings [22,62-64].

The itinerancy of homelessness and the high prevalence of mobile phone possession among homeless people has suggested a potentially important role for mobile health interventions targeting this vulnerable population [66]. However, our study underscores the difficulty that homeless individuals may have in retaining a single mobile device over a period of time sufficient to receive an intervention through it. Half of all 75 trial participants and only 40% of SmokefreeTXT arm participants still had their study-issued phone after 8 weeks. Qualitative interviews highlighted several challenges to successfully using these phones because of loss, theft, and problems with storage and recharging. As a result, efforts to develop mobile health interventions for homeless people will need to be coupled with innovations to help promote mobile device retention and use.

The negligible effect of both study conditions on complete 7-day smoking abstinence at any time point is disappointing and highlights the challenges to achieving long-term smoking cessation in this vulnerable group of smokers. This finding underscores that the optimal approach to promoting smoking cessation in this population remains uncertain but may ultimately require a combination of traditional and nontraditional intervention modalities targeting multiple levels of influence [16].

### Limitations

An important limitation of our study is that SmokefreeTXT was added to a relatively robust evidence-based tobacco treatment regimen of nicotine patch therapy and in-person counseling as well as frequent in-person abstinence monitoring. The high-contact nature of the study could have overwhelmed a small but meaningful treatment effect for SmokefreeTXT, which might be more optimally suited for smokers not already engaged (or able to be engaged) in more intensive treatment modalities. Another limitation is that participants in the control arm could have gained access to SmokefreeTXT without our assistance as it is free and publicly accessible, introducing the possibility of contamination. However, control arm participants were not told about this program, and surveys of control arm participants at 4 and 8 weeks found that none reported using an SMS text messaging program to support smoking cessation in the prior month. Other limitations of the trial include the small sample size and short duration of the study. Although our repeated-measures approach to outcome measurement offered adequate power to detect what we deemed to be a clinically important effect on CO-defined abstinence, we may have been underpowered to detect smaller treatment effects. Our qualitative interviews were wide-ranging in scope and not focused exclusively on SmokefreeTXT, and sampling was dictated by the number of trial participants in each arm who were willing to be interviewed rather than by attainment of thematic saturation. However, each of the identified themes emerged from more than 1 source, and later interviews generally did not uncover new thematic areas. Finally, our study was conducted at a large homeless health care program in Boston, so the findings may not be generalizable to other settings.

### Conclusions

SmokefreeTXT, added to nicotine patch therapy and in-person counseling, did not improve smoking abstinence in this 8-week pilot RCT for homeless smokers. Although program uptake was high, interaction with the program was minimal, and mobile device loss was common. Our qualitative findings suggest that future SMS text messaging interventions for this population should be better tuned to the unique circumstances of homelessness and coupled with innovative efforts to promote mobile phone retention over time.

## Appendix

Multimedia Appendix 1 CONSORT-eHealth checklist (V 1.6.1).

## Abbreviations

BHCHP: Boston Health Care for the Homeless Program
CO: carbon monoxide
GEE: generalized estimating equation
IQR: interquartile range
NCI: National Cancer Institute
OR: odds ratio
ppm: parts per million
RCT: randomized controlled trial
SMS: short message service
TTS: Tobacco Treatment Specialist
